# Impact of Field Insect Pests on Seed and Nutritional Quality of Some Important Crops: A Comprehensive Review

**DOI:** 10.1021/acsomega.4c08982

**Published:** 2025-02-28

**Authors:** Mohamed Ouaarous, Karim El Fakhouri, Noamane Taarji, Adil Baouchi, Moez Amri, Chaimae Ramdani, Mansour Sobeh, Abdelhalem Mesfioui, Mustapha El Bouhssini

**Affiliations:** † AgroBioSciences Program, College of Agriculture and Environmental Sciences, Mohammed VI Polytechnic University, Lot 660, Hay Moulay Rachid, 43150 Benguerir, Morocco; ‡ Laboratory of Biology and Health, Department of Biology, Faculty of Science, Ibn-Tofail University, Kenitra 14000, Morocco

## Abstract

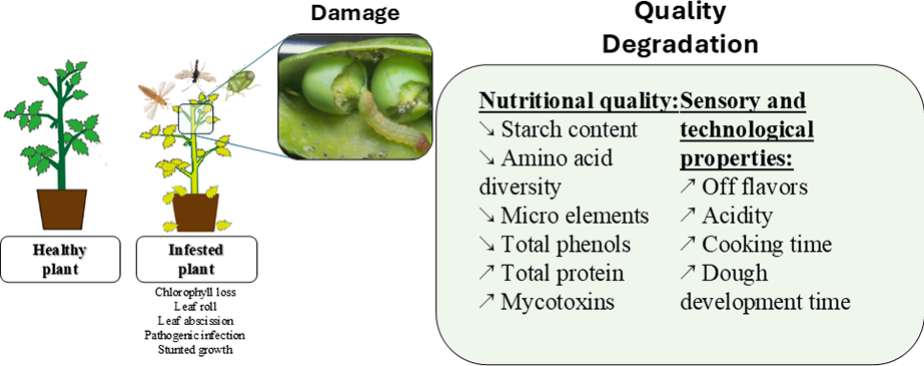

The increasing problem of insect pest infestation in agriculture and its impact on crop production and productivity require a thorough understanding and identification of efficient control solutions. This review explores the effects of insect infestations on crop productivity and seed quality with a focus on nutritional value, in particular, protein content, sugar levels, mineral composition, vitamin C concentration, antioxidant activity, and phenolic content. The paper compiles current studies on the diverse impacts of insect pests, including Hessian flies, Russian wheat aphids, Sunn pests, fruit flies, and others, on various crops such as wheat, soybeans, mung beans, durum wheat, bread wheat, olives, and citrus fruits. Integrated pest management (IPM) techniques including the implementation of digital technologies are highlighted as crucial methods for maintaining crop quality and ensuring food and nutritional security. This review provides more emphasis on seed morphological and nutritional quality characterization in response to insect pest infestation as well as the development of more practical methods for lowering crop quality losses and raising the nutritional quality of agricultural products by adopting a multidimensional approach for durable insect pest management.

## 1 Introduction

Malnutrition is a major social and economic issue with a global dimension. Over 2 billion people are estimated to be deficient in essential vitamins and minerals, including iron, zinc, iodine, and vitamin A.^1^ For decades, most developing countries have focused on increasing crop productivity and grain yield, often neglecting malnutrition-related issues. However, recent breeding efforts have shifted toward developing nutrient-rich food crops. This review specifically addresses the impacts of preharvest insect pest damage on crop quality, including alterations in nutritional composition, without delving into postharvest deterioration and storage-related damage, which have been comprehensively reviewed in other studies.

The impact of insect pests on agricultural productivity is anticipated to intensify, driven by the rising occurrences of abiotic stressors (drought, high temperature, cold, salinity) and biotic stressors including insect pests and diseases.^2^ Insect pests are the most detrimental biotic factors affecting agricultural productivity globally. According to Sharma et al, arthropods cause an estimated damage of 18–20% annual loss in agricultural productivity, translating into a potential loss of around US$470 billion.^3^ Furthermore, 15–20% of global vegetable production is lost annually due to field insect damage, with an additional 18–20% lost during storage.^4^

Climate change and weather disruptions significantly influence insect pest dynamics. Increased temperatures directly affect pest population dynamics, reproduction, survival, and distribution, as well as their interactions with natural enemies and the environment.^5^ In addition to causing direct damage by consuming leaves, fruits, and other plant parts, insects can cause indirect harm to plants by dispersing viruses, bacteria, and fungi leading to food contamination due to mycotoxins. Warmer environments promote rapid insect growth, resulting in more generations of insects per year and larger population sizes. According to refs (6 and 7), high temperatures frequently prevent plant defenses or insect/disease resistance, reducing grain production and quality. Thus, there is a significant risk of economic losses in agriculture alongside challenges to human food and nutritional security. In addition to climate-induced stressors, insect infestations impact crops by altering protein and amino acid composition, the availability of carbohydrates and lipids, and the organoleptic properties. Plant damage varies with the feeding type of the insect. Piercing, sucking insects that feed on plant vascular systems, like aphids, connect their specialized stylets to the phloem and cause minimum tissue disorder compared to other feeding habits, such as boring or mining, while chewing insects, like caterpillars, consume substantial amounts of plant tissues. Thrips use a combination of sucking and rasping to feed. Mining-type insect feed between epidermal cell layers within leaf tissues, which, via altering both the stomatal conductance and chlorophyll concentration, may result in a decrease in photosynthetic activity.^8−10^ Continuous feeding by any category of insect can lead to significant alterations in plant growth, development, and yield, manifesting as chlorosis, leaf loss, destruction, or defoliation, resulting in substantial losses of the commercial and technological quality of the crop.

Herbivore interactions with plants can trigger a series of physiological responses and activate both direct and indirect defense mechanisms, such as chemical and physical resistance. These interactions can also significantly influence various metabolic processes in the plant.^11,12^ Insects have developed various counterstrategies against plant defenses by releasing salivary chemicals called effectors, which interfere with and weaken the plant’s defense mechanisms. These effectors can suppress plant responses, but plants may recognize them through specific resistance proteins.^13,14^ Likewise, herbivory can result in symptoms such as systemic or localized chlorosis, aberrant gene expression, and nutrient transfer. In addition, salivary and midgut-regurgitated enzyme production performs a portion of the crop’s early enzymatic digestion. In cereal, Sunn pest *Eurygaster integriceps*, [Hemiptera: Scutelleridae] is a major cereal pest in the Central and West Asia, and North Africa (CWANA) region, severely affecting wheat and barley yields. The insect causes direct damage by feeding on foliage, stems, and grains and indirect damage by injecting digestive enzymes such as proteinase, lipase, and amylase. These enzymes degrade gluten, resulting in poor dough quality, reduced loaf volume, and an undesirable bread texture. The rapid dough relaxation also causes burning during baking, affecting the flavor. Therefore, breeding for insect resistance is essential to improve yield and maintain quality, focusing on developing varieties resistant to both biotic and abiotic stressors.^15,16^

For example, many international wheat breeding programs evaluate parent lines for traits such as potential yield, heat and drought resilience, insect resistance (against pests like aphids, Hessian flies, and Sunn pests), and enhanced nutritional quality. Tadesse et al. suggests that breeding for tolerance traits can help reduce the pressure on insect pests to develop resistance. Developing resistant varieties tailored to specific regions, combined with integrated pest management strategies, is essential for preventing the spread of aggressive pest biotypes.^17,18^

This review paper synthesizes available data on the effects of various field insect pest categories on the organoleptic, agromorphological, commercial, technological properties, and nutritional quality of some economically important food crops. The analysis is based on the feeding habits and host plant preference of the insect on some important crops under different farming and agroecological systems.

## 2 Bibliometric Analysis

**Figure 1:**
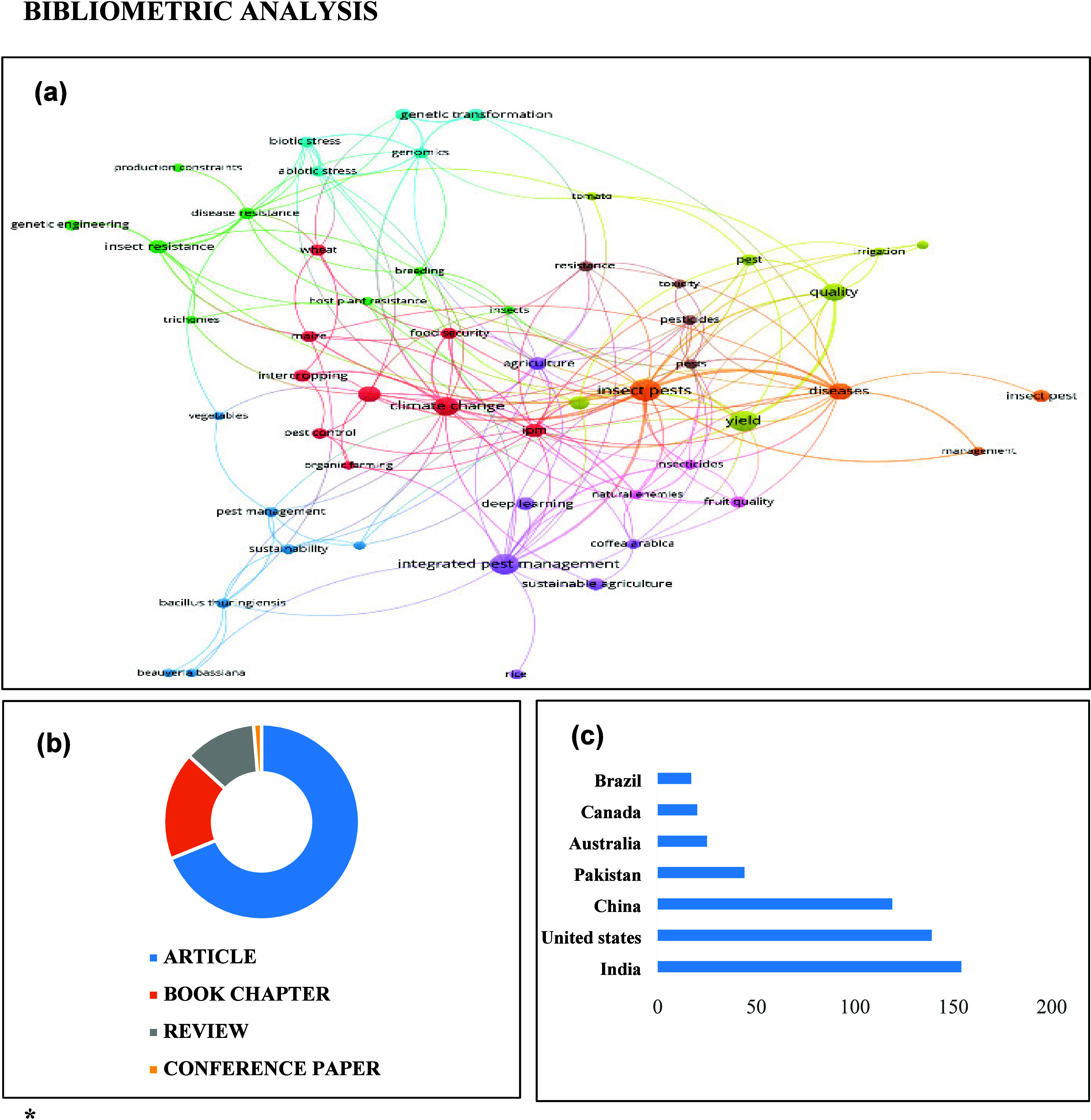
(a) Bibliometric network of keywords in publications on the impact of field insect on quality and yield losses. (b) Different types of papers published, (c) leading countries in publications assessing quality and yield losses by field insect pest.

The information in this review was obtained via bibliometric analysis using the Scopus, Google Scholar, and Web of Science databases. A network analysis using VOSviewer was used to highlight the connections between terms, providing a comprehensive overview of the state-of-the-art studies on the impact of field insect infestation on crop quality (Figure 1A). The search was conducted by using three keywords *“insect pest”, “yield”, “quality”,* resulting in a total of 642 documents. Most of them are original papers (62,8%), followed by book chapters (16.3%), reviews (10.9%), and conference papers (8.9%) (Figure 1B). Several bibliometric indices, including commonly used terms, were employed to conduct the network study. Furthermore, these findings made it possible to assess the most important published research. India had the highest number of documents (154), followed by the United States (139) and China (119) (Figure 1C).

### 2.1 Inclusion and Exclusion Criteria

Only full-text research publications published in the referred databases that assessed changes in sensory and nutritional quality resulting from insect infestation in the field until the end of 2023 were considered for this comprehensive review. Studies were excluded if they did not assess infestation levels, were books, unpublished articles, doctoral theses, commentaries, abstracts of conferences and congresses, or case reports, were not written in English or French, or were not quantitative studies.

## 3 Impact of Insect Infestation on Plant Growth and Physiological Changes

Insects from orders such as Lepidoptera, Homoptera, Hemiptera, Coleoptera, Orthoptera, Diptera, Thysanoptera, and Acarina are common crop pests. They cause direct damage by feeding on plant tissues, reducing growth and yield, and act as vectors for diseases, transmitting viruses, bacteria, and fungi.^19^

The impact of insect infestations on crop productivity is determined by constant and variable factors. Constant factors include specific pest development patterns and the type of damage, while variable factors encompass the timing and severity of damage, infestation duration, and environmental conditions. Less common damages include egg deposition, nest-making, and mutual interactions with other insects. Common feeding behaviors like chewing and sucking result in defoliation, fruit scarring, leaf mining, and stem damage.^20^

Boote et al. classify pest injuries into six functional categories: stand reducers, leaf-mass consumers, assimilate sappers, turgor reducers, architecture modifiers, and fruit feeders. These categories describe how pests impair plant physiology and yield, from reducing plant population through seed or seedling destruction to directly damaging reproductive structures. Each type of injury disrupts specific plant processes, such as photosynthesis, water regulation, or nutrient allocation, ultimately affecting crop productivity and quality.^21^

### 3.1 Types of Insect Feeding Damage and Their Impact on Plant Physiology

Insect pests with mouth parts designed for biting and chewing obtain food by biting plant fragments and chewing off the outer layers. Various types of damage are caused by this category, including the loss of photosynthetic tissues, defoliation in severe cases, destruction of buds and shoots, flowers, fruits, and seeds, disruption of sap flow, physical weakening of the stem, potential stem damage, and damage of seedlings and young plants. For example, chewing insects affect plant growth by inducing a defense mechanism in plants, which inhibits growth and reduces the rate of photosynthesis. Chewing insects transmit mouth secretions that lead to the defoliation of leaves, causing plants to increase their defenses. Plants that are bitten primarily experience phosphorylation cascades, electrical signaling, calcium ion fluxes, and system in release, which trigger the induction of the Jasmonate signaling pathway. Both the hexadecanoid and octadecanoid pathways are involved in jasmonate signaling. Plant growth is eventually limited because of the products of the Jasmonate pathway being lowered by the attack of a chewing insect or herbivore. In fact, by preventing development and triggering a defense mechanism, herbivores alter the metabolism of plants, inhibiting growth and reducing the rate of photosynthesis.^22^

Additionally, insect pests with piercing and sucking mouth parts can feed on the cell sap of plants, which hinders photosynthesis and devitalizes the plant; it causes external symptoms such as curling, yellowing, browning of leaves, stunted growth, and wilting in severe cases.^23^ Sap is extracted from either the phloem or xylem as well as from the general tissues of leaves, roots, or fruits. The various types of damages caused by pests include damage caused by boring and leaf miners, damage caused by gall making, feeding on roots and tubers, damage caused by nest making, and damage caused by mutualism where pests indirectly benefit from mutualistic relationships with other organisms, leading to plant damage.^24^

### 3.2 Pests as Vectors of Plant Pathogens

The impact of pests that transmit pathogens is significant as even a small number of infected individuals can lead to widespread and severe disease outbreaks, posing major risks to plant health and crop production. Pests that act as vectors of pathogens can cause damage in two ways. Indirect vectors are insects that create feeding punctures or rupture the protective epidermis while feeding or laying eggs. Subsequently, these punctures become infected with fungal and bacterial spores. Direct vectors are insects that are commonly referred to as “biological vectors”. They are responsible for actively transmitting as they often act as intermediate hosts. Insects serve as vectors for various plant pathogens, such as viruses, bacteria, nematodes, fungi, and Oomycota (Figure 3).^25^ These pathogens are responsible for causing plant diseases that have a substantial impact on crop yield and quality.^26^

Both field insect pests (European corn borer) *Ostrinia nubilalis* [Lepidoptera: Crambidae] and (corn earworm) *Helicoverpa armigera* [Lepidoptera: Noctuidae] significantly contribute to fungal infections in crops by damaging plant tissues, which serves as entry points for pathogens such as *Fusarium verticillioides*. This fungus produces mycotoxins, particularly fumonisins, which degrade grain quality and pose health risks to consumers. Larval feeding facilitates fungal spore spread through direct contact with fecal pellets, leading to contamination even in seemingly intact kernels. Studies have shown that infestations by these pests increase the prevalence of mycotoxins in maize crops, correlated with the severity of pest damage. This highlights the intertwined roles of insect pests and fungal pathogens in reducing the safety and nutritional value of agricultural produce.^27,28^

### 3.3 Plant Defense Mechanisms and the Complex Dynamics of Plant-Herbivore Interaction

Plants respond to herbivory using three main categories: nonpreference (or antixenosis), antibiosis, and tolerance through various morphological, biochemicals, and molecular mechanisms to mitigate the impacts of herbivore attacks.^17^ Plants can exhibit phenotypic plasticity, altering their genotype frequencies, phenotypic traits, and plant microbiota to increase resistance to herbivores.^29^ These responses are mediated by physical barriers such as waxy cuticle layers, spines, and trichomes, as well as chemical shields involving plant secondary metabolites (SMs) and volatile organic compounds (VOCs).^30^ Plant responses to herbivory start with the acquisition of physical stimuli and chemical substances, which then trigger signal transduction, activation of hormones (jasmonic acid, salicylic acid, and ethylene), and the emission of volatile organic compounds (VOCs) and secondary metabolites (SMs).^31^

The effects of herbivory on plant interactions with florivores during flowering can be influenced by the type of herbivory (root or leaf) and result in changes in phytohormone profiles and secondary chemistry in buds and flowers.^32^ During maturation, insect infestation can have various effects on plant maturation. For example, feeding by stink bugs can induce delayed senescence in soybean stems, leaves, and pods, resulting in delayed pod maturation.^33^ However, under appropriate conditions, some insect pests can enhance the productivity of some crops. There is a phenomenon known as the “pruning effect” that occurs when an insect feeds, limiting the growth of one organ and increasing the size or weight of others. Field bean yields can be increased by minor *Aphis fabae* [Hemiptera: Sternorryncha] infestation, most likely due to a reduction in apical growth.^34^ Understanding the correlation between pest or injury levels and crop yield can be quite intricate, especially when considering factors such as compensatory growth in surviving plants or the variability of resistance to attacks over time. Identifying the causes of decreased yields becomes quite evident when the plants are attacked or destroyed. Understanding the factors that contribute to the decreased yield can be challenging, especially when plants or yield-forming organs are not destroyed. However, crop physiology can provide some valuable insights into these processes.

## 4 Impact of Insect Pest Infestation on the Nutritional Value of Different Crops

Several factors directly or indirectly influence the nutritional quality of crops, including soil pH, nutrient availability, texture, organic matter concentration, and soil-water interactions, in addition to climate factors like temperature, rainfall, and light intensity. Furthermore, important factors also include cultivar and type of crop, postharvest management and storage, fertilizer treatments, and cultural methods. In addition to abiotic stresses, insect pest infestations can have a substantial influence on the nutritional value of agricultural output. The effects of infestation differ based on the type of insect, type of damage, host plant, growth stage, infestation degree, and the adopted control measures. In general, infestation can cause changes in carbohydrate and fat content, increase in protein content, and reduce essential amino acid and fiber levels (Table 1). Overall, insect infestation can have a detrimental effect on the nutritional value, emphasizing the need for appropriate pest management measures.^35^ The insect species selected for this review are the only ones documented in the literature for their direct impact on the nutritional quality of crops, with numerous studies highlighting their effects on both seed composition and overall quality.

**Table 1:** Impact of Insect Pests’ Infestation on Nutritional and Technological Quality of Different Crops

insect (common name: scientific name)	feeding habit	target crop	effect on quality	ref
**Stink bug:*****Euschistus servus***	Sap feeders	Soybean	- Seed coat mottling	(37)
			- Seed shrinkage	
			- Increased palmitic, stearic, and oleic acids	
			- Decreased linoleic and linolenic acids	
			- Increased protein content	
**Spotted pod borer:*****Maruca vitrata***	Chewing insects	Mung bean	Increase in levels of	(40)
			- Protein (23.44%)	
			- Amino acids (0.130%)	
			- Total sugar (1.38 mg/g)	
			- Reducing sugar (0.59 mg/g)	
			- Nonreducing sugar (0.79 mg/g)	
**Hessian Fly:*****Mayetiola destructor***	Chewing insect	Durum wheat	Decrease of:	(45)
			- 61.1% in grain yield	
			- 13.6% in test weight	
			- 3.6% in flour yield	
			Increase of	
			- 11.9% protein content	
			- 3.7% alkaline water retention capacity	
			- 3.1% kernel softness	
**Russian Wheat Aphid:*****Diuraphis noxia***	Sap sucking	Wheat	- Decreases in dough strength	(56)
			- Shortened farinograph dough development time.	
**Sunn Pest:*****Eurygaster*****spp.**	Chewing insect	Bread wheat	- Decreased protein content per kernel by 27% in bread wheat and 20% in durum wheat.	(63)
		Durum wheat	- Decreased bread quality with 20% damaged kernels	
**Wheat Stem Sawfly:*****Cephus pygmaeus*****L.**	Chewing insect		- Decrease of protein from 13.22% in healthy grains to 12.65% in wheat stem sawfly infested kernels	(64, 67)
**Mediterranean Fruit Fly:*****Ceratitis capitata***	Chewing insect	Citrus	- Decrease in lactose, maltose, and glucose content by more than 50%.	(71)
			- Decrease of 38% to 99% for calcium	
			- Decrease of 39% to 86% for phosphorus	
			- Complete depletion of copper, zinc, and iron levels	
**Oriental Fruit Fly:*****Bactrocera dorsalis***	Chewing insect	Citrus	- Decrease of vitamin C from 27.81 μg/mL in healthy fruits to 18.65 μg/mL in infested oranges	(72)
			- Decrease in antioxidant activity	
			- Decrease in total phenol content from 56.267 mg GAE/g, to 40.137 mg GAE/g	
			- Decrease in the free-radical scavenging activity from 27.69% in healthy orange juice to 5.39–19.08% in infested ones	
**Olive Fruit Fly:*****Bactrocera oleae***	Chewing insect	Olive	- Increase of free acidity from 0.6% to 3.4%	(73)
			- Decreased levels of polyphenols from 268.51 to 13.52 mg kg-^1^ gallic acid equivalent	
**Coffee Berry Borer:*****Hypothenemus hampei***	Chewing insect	Coffee	- Slow and irregular roasting	(75)
			- Reduced aroma and flavor	
			- Increased bitterness	
			- Development of off flavors (chemical, tarry, moldy, burnt)	
			- Ochratoxin contamination	

### 4.1 Pulses

#### 4.1.1 Stink Bugs

Stink bugs have a significant impact on the quality of soybean seeds, making them the most significant insect pests in this context. The southern green stink bug, green stink bug, and brown stink bug scientifically known as *Euschistus servus* [Hemiptera: Pentatomidae] are the most common stink insect species found in soybean fields in the United States. McPherson et al.^36^ reported that *E. tristigmus*, *E. variolarius*, and *Piezodorus guildinii*, [Hemiptera: Pentatomidae] are potential pests in some regions. Stink bugs prefer consuming young and sensitive reproductive tissues and developing seeds, but may also feed on plant stems, leaves, blossoms, and seeds.^36^ Their feeding mechanism consists of penetrating and sucking plant tissue with their mouthparts. Damage is caused by a variety of variables, such as fruit abortion, seed harm, the reduction of plant fluid, and an injection of enzymes for digestion. The pods may become infected at the feeding site by a variety of fungi and bacteria, leading to either localized or widespread pod degradation. Severe injury to seeds raises the amount of protein, increases the proportion of tiny seeds, decreases the oil content, and increases the amount of moldy or discolored seeds.^37^ Grain that has been stored normally deteriorates due to increased moisture content, splits, fractures, and destroyed seed coatings; however, over time, major harm from feeding stink bugs can cause more serious storage damage.^38^

#### 4.1.2 The Spotted Pod Borer

Spotted pod borer, *Maruca vitrata* [Lepidoptera, Crambidae], is among the harmful lepidopteran pests in food legumes and is recognized for its wide host range and high level of impact, occurring from the seedling to pod formation stage and global distribution. According to Roufi and Sardar,^39^*M. vitrata* larvae inflict huge damage on all plant growth stages, from seedling until pod production, with the flowering period being particularly vulnerable. They consume stems, peduncles, blooms, and pods of different food legumes crops. Among the ten mung bean (*Vigna radiata*) varieties that were evaluated, the sensitive cultivar exhibited higher levels of protein content (23.44%), amino acids (0.130%), total sugar (1.38 mg/g), reducing sugar (0.59 mg/g), and nonreducing sugar (0.79 mg/g) compared to the highly tolerant cultivar LGG-497.^40^ Additionally, phenols in the resistant cultivar were higher (21.03 mg/g) than in the susceptible cultivar (20.00 mg/g).^39^ According to Halder et al.,^40^ there was a negative association between the phenols content and pod damage and a strong positive relationship between protein, amino acids, reducing and nonreducing sugars, total sugar, and pod damage.

### 4.2 Cereals

#### 4.2.1 Hessian Fly

*Mayetiola destructor* (Say) [Diptera: Cecidomyiidae], known as The Hessian Fly, poses a significant threat to wheat (*Triticum* spp.) cultivation across several regions such as North Africa, United States, Western Asia, and southern Europe.^41−43^

**Figure 2:**
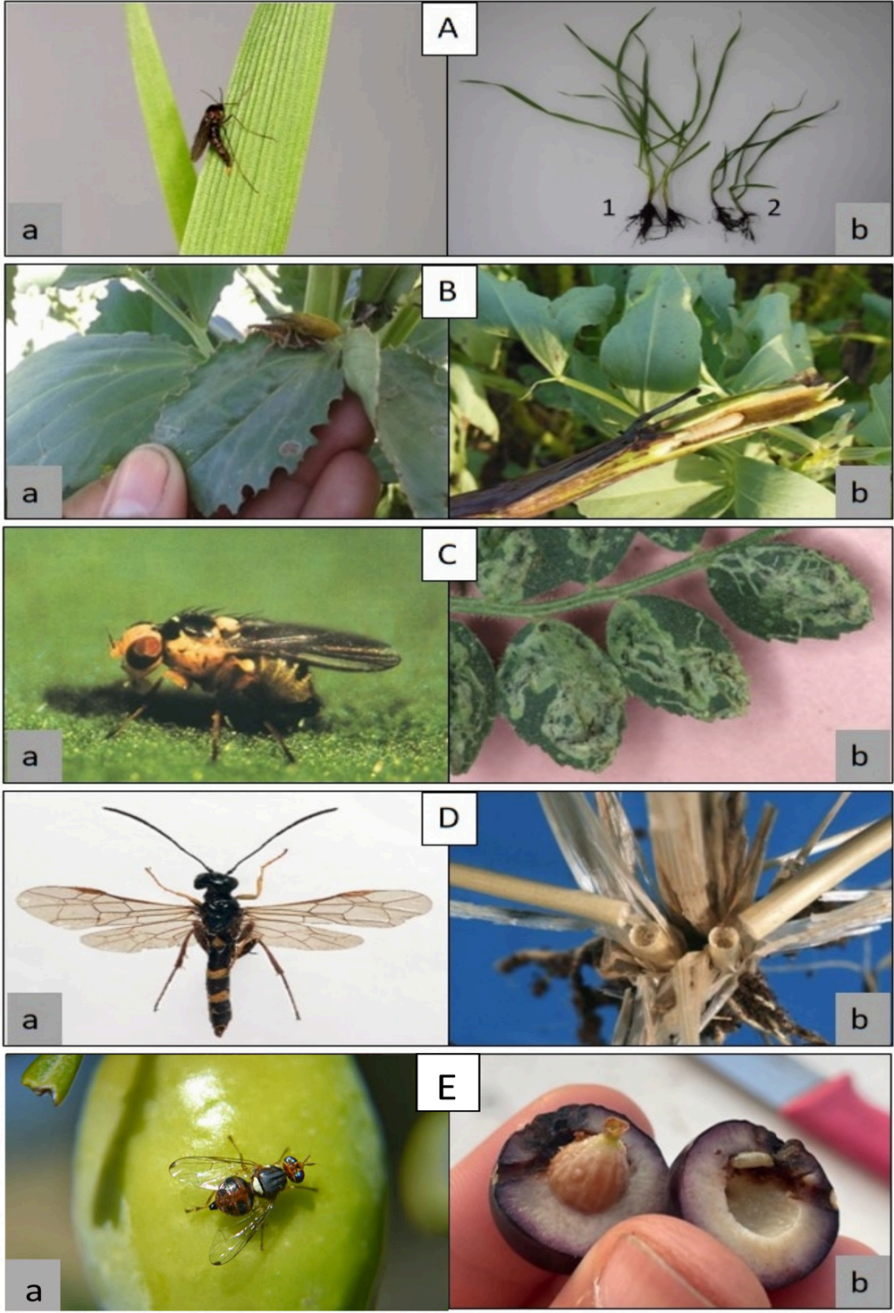
Different insect pests (left) and associated damage (right): Adult Hessian fly (HF), *Mayetiola destructor* (left) **A.a**/ resistant wheat **A.b.1**, susceptible wheat **A.b.2**. Fava bean Stem Borer (FBSB), *Lixus algirus* L. (left) **B.a**/ FBSB larva boring stem (right) **B.b**. Adult Chickpea Leafminer, *Liriomyza cicerina* (left) **C.a**/ Leaf mining damage in chickpea leaflets (right) **C.b**, Wheat Stem Saw Fly (WSSF) adult, *Cephus cinctus* (left) **D.a**/ Stems cut by WSSF (right) **D.b**. Adult Olive Fruit Fly *Bactrocera oleae* (left) **E.a**/ Infested Olive Fruit (oviposition and larvae feeding) (right) **E.b**.

The Hessian fly is a significant insect pest of wheat in Morocco, resulting in average production losses for durum and bread wheat of 32 and 36%, respectively.^42^ Berzonsky et al.^44^ estimate $100 million in yearly US damages from these infestations. The feeding of first-stage (L1) larvae of the Hessian fly results in the stunting of young wheat stems (Figure 2), which become green and stop growing.^42^ According to Buntin,^45^ Hessian fly infestations reduce grain wheat quality and quantity. Hessian fly susceptible cultivars were evaluated for grain test weight, flour yield, grain protein content, alkaline water holding capability, and kernel softness. The authors found that test weight loss significantly increased when autumn infection levels increased beyond 40% of infected tillers. At early planting, Hessian fly damage resulted in a significant 76.9% and 61.1% reductions in grain yield, accompanied by a 20.9% decrease in test weight and a 5.4% decline in flour yield. In contrast, it resulted in increases of 2.9% in kernel softness, 3.2% in alkaline water retention capacity, and 10.9% in protein content. Likewise, Hessian fly damage at the suggested planting date resulted in a 61.1% decrease in grain yield and a 13.6% reduction in test weight, whereas protein content, alkaline water retention capacity, and kernel softness improved by 11.9%, 3.7%, and 3.1%, respectively, and a 3.6% decrease in flour output. Despite the significant impact of the planting date on grain quality parameters, it was noted that Hessian fly infestations could lead to a deterioration in the quality of affected grain, rendering it unsuitable for milling and baking purposes.

In another study, Saltzmann et al.^46^ observed that biotype L larvae increased free amino acids for susceptible plants compared to resistant ones. Three necessary amino acids (histidine, methionine, and phenylalanine) that insects cannot produce and must get in their diet were among those that increased in abundance in susceptible plants.

#### 4.2.2 Russian Wheat Aphid

Russian wheat aphid (RWA) *Diuraphis noxia* (Kurdjumov) [Hemiptera: Aphididae], originally from Central Asia, is currently widespread throughout the world, with a recent detection in Australia. It can be found in various regions including southern and central Europe, the Middle East, North and South Africa, as well as in the USA and Canada.^44,47^

Among cultivated crops, wheat and barley are the most susceptible to RWA, followed by rye, triticale, and oats.^48^ This insect is characterized by its piercing-sucking mouthparts, which it uses to extract sap from its host plant. During feeding, it injects toxic saliva that disrupts the photosynthetic process, resulting in chlorosis, leaf curling, and the formation of white, purple, or yellowish streaks along the leaves.^49−51^ These symptoms may result in an enormous decrease in biomass and, in extreme circumstances, plant death.^49,50^ Yield losses due to the Russian aphid in southern Africa from 21 to 92%^52^ and between 25 and 60% in Turkey.^53^ In Ethiopia, losses have been estimated at 68%,^54^ and in Kenya losses have been estimated at 90%.^55^

However, it is worth noting that RWA infestations can also affect the quality of wheat. Butts et al.^56^ found that RWA infestation had no effect on milling characteristics such flour yield and starch damage or grain hardness, as measured by the particle size index. However, unsprayed plots with infested wheat plants had a modest rise in flour ash amount compared to noninfested ones. According to the same study, the presence of Hessian fly infestations can have an impact on the strength of the dough mix and the dough development time. They noted significant decreases in dough strength, indicated by the Zeleny-sedimentation volume and shortened farinograph dough development time, in samples affected by Hessian fly infestation. Additionally, it was observed that flour water absorption remained unaffected by the infestation due to consistent levels of protein content and starch damage.

#### 4.2.3 Sunn Pest

The Sunn pest, also known as the suni bug (*Eurygaster* spp.) [Hemiptera: Scutelleridae], is a serious pest of wheat found in Turkey, eastern Europe, the near and middle east, and North Africa.^57−60^ This pest is widespread in these regions and causes significant damage to wheat crops. The Sunn pest pierces grains, degrading gluten and affecting flour baking properties, resulting in reduced loaf volume and altered texture of bread. The severity of damage varies among wheat varieties, with different reactions observed based on grain type and color.^60−62^ Sunn pest attacks on wheat result in significant quality and yield losses, reducing test weight, protein content, sedimentation value, and increasing flour ash content.^63^ According to this study, protein content decreases with increasing Sunn pest damage, and the sedimentation value reflects reduced gluten strength. For instance, a variety such as Gerek 79 showed an average loss of 5.4% in protein content when compared with Atay 85, which showed the least degree of damage.

Sunn pest infestation can also impact the sedimentation value (SV), a crucial parameter used to evaluate the quality of wheat protein, and the gluten strength essential for determining baking quality. A decrease in SV indicates a shift from strong to weak gluten, which can significantly affect the quality of the bread. In the same study by Kınacı et al.,^63^ it was observed that the sedimentation value (SV) decreased across all groups as Sunn pest damage increased.

In a study conducted by Hariri et al.,^1^ it was demonstrated that higher infestation levels by the Sunn pest resulted in a notable reduction in protein content in wheat by approximately 2.8% in bread wheat and 1.9% in durum wheat lines. Furthermore, the protein content per kernel dropped significantly, with 27% in bread wheat and 20% in durum wheat due to insect damage. The study also revealed that the quality of two-layered flatbread baking is more tolerant of minor damage to the kernels. However, when 10% or more of the kernels are damaged, there was a deterioration in bread quality on bread quality, making it impossible to produce satisfactory bread once 20% of the kernels were affected.

#### 4.2.4 Wheat Stem Sawfly

The wheat stem sawfly, *Cephus pygmaeus* L [Hymenoptera: Cephidae], is native to Europe and has spread to other cereal-growing regions worldwide, including the Middle East, Central Asia, Turkey, Iran, Iraq, Cyprus, Algeria, Lebanon, Palestine, Egypt, and Morocco.^44,64^

The larva feeds on the stem, destroying the vascular tissues and hindering the sap circulation (Figure 2). Seeds from attacked stem spikes are poorly nourished and underdeveloped, leading to a reduction in their quality.^65^ In Canada, average economic losses of $27.45 per hectare have been reported by Beres et al.^66^ In Morocco, yearly yield losses due to this pest are around 15%.^62^

According to Holmes,^67^ infestation by the wheat stem sawfly can indeed impact the quality of wheat. In his study, it was found that larvae of the wheat stem sawfly decreased the protein content by an average of 0.6% in Thatcher bread wheat variety, with a maximum reduction reaching 1.2%. Ozberk et al.^64^ reported a decrease in protein levels from 13.22% in healthy grains to 12.65% in infested kernels.

### 4.3 Fruit

#### 4.3.1 The Mediterranean Fruit Fly

The Mediterranean fruit fly (or medfly), *Ceratitis capitata* (Wiedemann) [Diptera: Tephritidae], is native to Africa and has a global range that includes many tropical, subtropical, and temperate climates. The multivoltine polyphagous species may feed on over 300 host plants, making it one of the most significant fruit pests globally.^68^ Adult oviposition, which enhances the spread of fungi and bacteria, and larval feeding activity are responsible for fruit destruction (Figure 2).^69^ The insect can cause fruits to ripen more quickly, modify their peel color, affect the nutritional content of juices, and deteriorate the pulp.^70^

According to Onovughakpor et al.,^71^ the medfly infestation resulted in a significant decrease in the crude protein content when compared to fruits that were not attacked. Furthermore, an analysis of the carbohydrate content of infested fruits revealed a distinct trend. Following single- and double-point attacks per fruit, the values of saccharose, maltose, and glucose exhibited an increase, while lactose experienced a significant decrease. Overall, infestation by *C. capitata* resulted in a reduction in lactose, maltose, and glucose content by more than 50% in the attacked citrus fruits, indicating substantial alterations in the nutritional profile of the fruits because of medfly attacks.

Furthermore, the infestation by *C. capitata* resulted in a considerable reduction in both calcium and phosphorus content within the attacked fruits, with reductions ranging from 38% to 99% for calcium and from 39% to 86% for phosphorus. Additionally, the microelements of zinc, iron, and copper, in the infested fruits were completely depleted, reaching zero. These findings highlight the substantial impact of *C. capitata* infestation on the mineral content of citrus fruits, underscoring the need for further investigation into the mechanisms underlying these depletions and their potential implications for fruit quality and nutritional value.^71^

**Figure 3:**
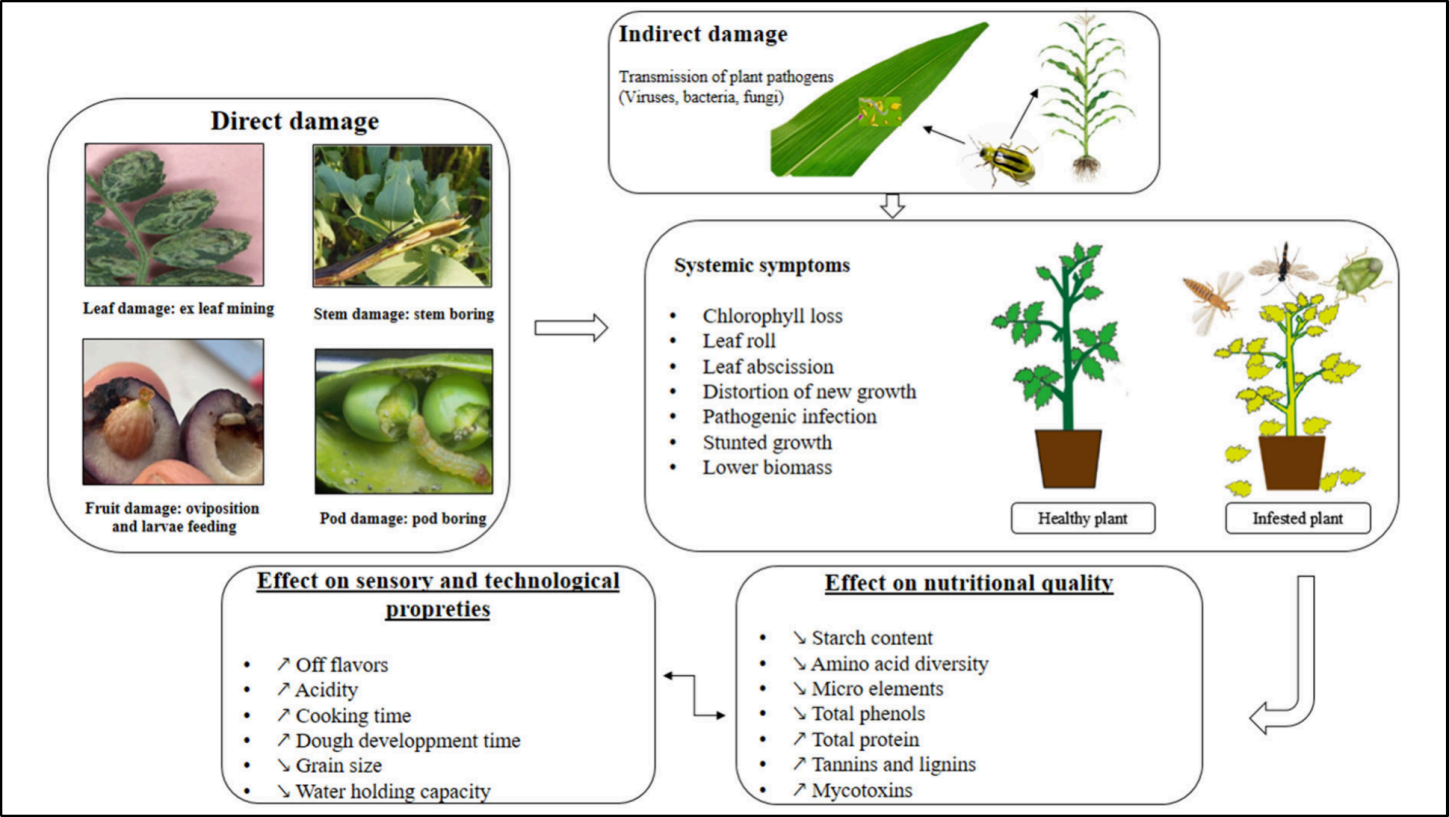
Insect injury can occur through the direct feeding and indirect transmission of plant pathogens.

#### 4.3.2 The Oriental Fruit Fly

*Bactrocera dorsalis* [Diptera: Tephritidae] is a harmful insect that feeds on various fruit crops. It is widely distributed, being present in over 65 countries, including regions in America, Oceania, and most of continental Africa (sub-Saharan countries). In addition, it is considered a quarantine pest in other parts of the world. Female fruit flies deposit their eggs in ripe fruits while they are still attached to the tree, and the resulting larvae make the fruit unsuitable for consumption, causing significant deterioration before and after harvesting.^72^

According to Ni et al.,^72^*B. dorsalis*-infested oranges had lower vitamin C (Vc) concentrations than controls. The concentration of Vc in orange juice decreased to 18.65 g/mL after 10 days of egg introduction (with larvae removed) compared to that in healthy orange juice (27.810 μg/mL). The antioxidant activity was negatively impacted by this decrease in vitamin C concentration. In addition, the total phenol content of orange juice was significantly reduced following *B. dorsalis* infestation. The average total phenol content of healthy orange juice recorded a higher value with 56.267 mg GAE/g, compared to the infested ones ranging from 9.748 to 40.137 mg GAE/g, 10 and 3 days after egg inoculation, respectively. The free-radical scavenging activity of orange juice decreased significantly in infested samples, ranging from 27.69% in healthy orange juice to 5.39–19.08% in infested ones, which indicates strong antioxidant activity. The removal of larvae from juice samples, as demonstrated by earlier analysis, had a significant effect on the orange juice’s free-radical scavenging rate, emphasizing the pest infestation’s impact on the antioxidant activity of the juice.^72^

#### 4.3.3 The Olive Fruit Fly

The olive fruit fly, *Bactrocera oleae* (Rossi) [Diptera: Tephritidae], is an insect that causes significant harm to olive crops all over the world, particularly in North America and the Mediterranean region. The *B. oleae* adult females deposit their eggs directly below the olive epicarp, giving the larvae instant access to nutrients upon its emergence, altering oil quality by reducing phenol concentration and antioxidant levels.

The presence of the olive fruit fly has been shown to have a substantial effect on the free acidity of olive oil derived from the Chemlali cultivar.^73^ The investigation revealed a direct association between the free acidity of olive oil and the extent of fly infestation. The free acidity values exhibited an important increase, rising from 0.6 to 1.5 and 3.4, in conjunction with infestation levels of 10%, 15%, and 100%, respectively. The existence of emergence holes created by the olive fruit fly enables the entry and growth of bacteria and fungi, resulting in an increase in acidity caused by the hydrolytic enzymes and lipolytic activity of these microorganisms. The presence of flies caused significant alterations in the phenolic composition of the olive oil. The study observed a decrease in the overall phenolic concentration when the olives ripened and were infested by olive fruit flies. Throughout the experimental period, the levels of polyphenols exhibited a decline, ranging from 268.51 to 13.52 mg kg^–1^ gallic acid equivalent. The invasion of the olive fruit fly led to substantial reductions in the overall quantity of phenolic compounds in oils derived from fully mature olives. The decrease in the phenolic concentration became apparent as the percentage of fly infestation rose. For example, olive oil extracted from olives with an attack percentage exceeding 50% consistently exhibited phenolic content levels below 75 mg kg^–1^ of olive oil. The decrease in the level of phenolic compounds was linked to variables such as the activity of polyphenoloxidase, which catalyzes changes resulting from larval damage to tissues. Additionally, the exposure of olive pulp to oxygen and external influences through exit holes formed by the olive fruit fly contributed to this decline. According to this study, an increase in infestation levels resulted in a decrease in phenolic content, which had an impact on the sensory qualities of the oil.^73^

#### 4.3.4 The Coffee Berry Borer

*Hypothenemus hampei* Ferrari [Coleoptera: Curculionidae, Scolytinae], also known as The Coffee Berry Borer (CBB), which is native to Africa, is considered the most significant pest that has a negative impact on the quality of coffee beans. It is prevalent in primary coffee-growing regions of the world. Even at low infestation levels, CBB can deteriorate the quality of the coffee beans. Arabica and Robusta are also targets for CBB, even though Robusta was probably its first host.^74^ CBB causes an estimated US$0.5 billion in economic loss annually. This encompasses both decreases in output due to berries falling early and the reduced market value of bored harvested berries. Thus, the Coffee Berry Borer threatens worldwide coffee quality and economic value.

The Coffee Berry Borer (CBB) significantly affects coffee quality through both direct and indirect mechanisms. Directly, CBB causes harm to coffee beans by penetrating them to deposit eggs, resulting in the creation of galleries. This damage is visually identifiable by one or more clean-cut circular holes in the beans, which can give the bean a ragged appearance if the attacks are heavy. Such physical damage directly impacts the sensory quality of coffee, as damaged beans are classified as “insect-infested” or “insect-damaged” according to ISO standards, indicating a medium influence on the sensory quality of beans.^75^

The presence of CBB and the galleries they form indirectly facilitate the colonization of beans by several fungal species, such as *Fusarium* spp. and *Aspergillus ochraceus*. *A. ochraceus* is especially alarming, because it generates ochratoxin A, which is a mycotoxin that is both nephrotoxic and carcinogenic. The presence of ochratoxin A in coffee beans not only poses health risks but also affects the sanitary quality of coffee, as it is regulated under European Union standards for roasted and soluble coffee.^76^ On the other hand, the CBB damage causes multiple changes to the coffee bean roasting process. Beans damaged by CBB have been reported to roast irregularly, often resulting in a darker color after roasting. This irregular roasting is due to physical damage to the beans, which can alter their density and moisture content, leading to uneven heat absorption during roasting. Galleries formation by the CBB in the beans allows subsequent infections by bacteria and fungi and weakens the structural integrity of the beans, leading to uneven roasting. This can have a significant impact on the flavor profile of the coffee as the degree of roast is intricately linked to the development of flavor compounds. Consequently, the presence of insect-damaged beans can lead to a batch of coffee with inconsistent flavor quality, affecting the overall sensory experience.

## 5 Plant Breeding and Integrated Pest Management Strategies to Minimize the Impact of Insect Pests

### 5.1 Biofortification

Traditional breeding efforts have historically focused on developing cultivars suitable for mechanical harvesting with high yield, large size, and resistance to diseases, often neglecting their nutritional quality. This lack of attention toward enhancing nutritional value can be attributed to limited understanding of metabolic pathways and their genetic components, which hindered progress in devising strategies to enhance the nutritional quality of crops. There has been a change in attention toward the cultivation of crops that are rich in nutrients, in order to meet the consumer demand for high quality and nutritious foods. Biofortification, which involves enhancing the nutritional quality of crops, encompasses three primary strategies: conventional biofortification, transgenic biofortification, and agronomic biofortification.^77^

Biofortified crops provide essential nutrients such as iron, zinc, vitamin A, and folate. These nutrients are often lacking in the diets of vulnerable populations, making biofortified crops a sustainable solution. These biofortified varieties in sweet potato, cassava, maize, bean, pearl millet, rice, and wheat were specially developed to increase both the nutritional value and the ability to resist to abiotic stresses (drought, heat, cold, acid soil) and several biotic stresses including mostly disease, viruses, bacteria and few cases for insect pests, which might potentially enhance agricultural output and increase the income of smallholder farmers^78^ (Table 2). A few research studies have investigated the efficiency of biofortified crops in controlling insect pests. Patange et al.^79^ showed that the zinc and iron biofortified pearl millet genotype AHB-1200 recorded the least population of pests viz., shoot fly (*Atherigona approximate* Mall.) [Diptera: Muscidae], shoot borer *Chilo partellus* [Lepidoptera: Crambidae], ear head borer *Helicoverpa armigera* [Lepidoptera: Noctuidae], flower chafer beetle (*Oxycetonia versicolor* Fabricius and *Chiloloba acuta* Wiedemann), and hairy caterpillars (*Amsacta* sp.) by virtue of its genetic potentiality and maximum population of natural enemies. Wang et al.^2^ revealed that the simultaneous use of Zn fertilizers in the form of ZnSO4 through foliar application, along with insecticides such as Acetamiprid and Imidacloprid in winter wheat increased grain yield by 3.7–4.9%, improved Zn concentration in grains, and effectively controlled wheat aphids (*Macrosip humavenae*).

**Table 2:** Some Biofortified Crops Have Resistance to Major Biotic Stresses

crop	cultivars	nutrient	category of biofortification	countries of first release	biotic trait	ref
**Sweet potato**	Two cultivars, NASPOT 12 O (Namulonge sweetpotato 12 orange-fleshed) and NASPOT 13 O (Namulonge sweetpotato 13 orange-fleshed)	Provitamin A	Conventional breeding	Uganda	Resistance to sweet potato virus disease (SPVD) and *Alternaria bataticola* blight	(82)
**Pearl millet**	Open-pollinated varieties (Dhanashakti) and hybrids (ICMH 1202, ICMH 1203, and ICMH 1301)	Iron, zinc	Conventional breeding	India	Resistance to blast and downy mildew diseases	(83, 84)
**Pearl millet**	AHB-1200	Zinc and iron	Agronomic biofortification	India	Resistance to shoot fly, shoot borer, ear head borer, flower chafer beetle, hairy caterpillars	(79)
**Cassava**	The Nigerian cvs TMS 95/0505 and TMS 91/02324	Provitamin A	Transgenic (RNAi-mediated technology)	Nigeria, Democratic Republic of Congo	Resistance to cassava mosaic disease (CMD) and cassava brown streak disease (CBSD)	(85)
**Maize**	Obatanpa GH’	Provitamin A	Conventional breeding	Ghana	Resistance to the Maize streak virus (MSV), lowland rust incited by *Puccinia polysora* and resistance to blight, caused by *Bipolaris maydis*	(86)
**Rice**	dhan62 and DRR Dhan 63 are a zinc-biofortified rice varieties and biofortified Iron Rice (BIR1)	Zinc	Conventional breeding	Bangladesh	Resistance to blast disease and to bacterial blight	(87)
**Wheat**	BHU 1 and BHU 6	Zinc	Conventional breeding	India	Rust and foliar disease resistant	(88)
**Wheat**	Cultivar“’Liangxing99”’ is Winter wheat (*Triticum aestivum*)	Zinc	Agronomic biofortification	China	Wheat aphid (e.g., *Macrosip humavenae*)	(89)

In addition, several studies have confirmed the significant effect of enhancing the nutritional content of wheat, rice, and common beans through the use of foliar Zn fertilizers. These zinc fertilizers can be applied simultaneously with fungicides and insecticides to manage foliar diseases, such as leaf rust, and insect pests, such as aphids. This approach increases the Zn concentration in grains, reaching optimal levels for human nutrition.^80^

Nevertheless, the potential benefits, biofortification faces challenges in gaining public acceptance, particularly with transgenic approaches.^81^

### 5.2 Adoption of Integrated Pest Management

Over time, pest control concepts have evolved into pest management, emphasizing the utilization of agro-ecosystem features and farmers’ management abilities to integrate various strategies and practices for cultivating healthy and profitable crops, while reducing the reliance on agrochemical inputs (IAPPS, 2024).^90^

**Figure 4:**
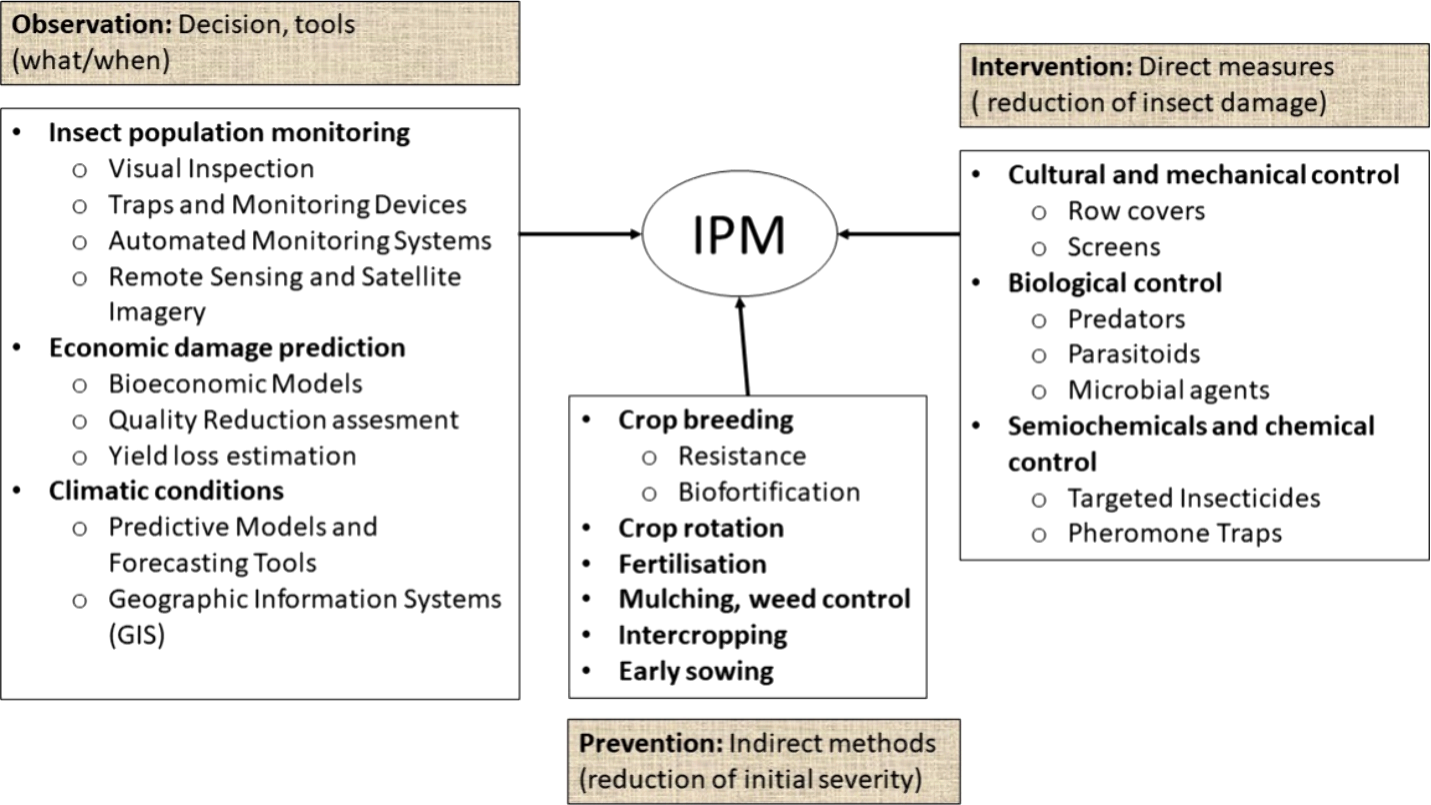
Main practices within integrated pest management (IPM) used to reduce impact on the nutritional quality and yield components.

Multiple control options can minimize quality and yield losses caused by insect pests, such as biological control, host plant resistance, cultural control, behavioral control, physical or mechanical control, chemical control, and microbial control (Figure 4). These strategies aim to prevent pests from reaching levels that result in economic damage.^91,92^

#### 5.2.1 Host Plant Resistance Breeding

Host plant resistance (HPR) is a vital pest control strategy, particularly in developing countries, where pesticide use may be limited due to feasibility or safety concerns. Resistant crop varieties offer advantages such as ease of use, cost-effectiveness, broad spatial and temporal effectiveness, and reduced environmental impact.^93^ Recent advances in biotechnology, molecular breeding, and omics have improved the implementation of HPR, originally conceptualized by Painter.^94^ The process involves four stages: screening germplasm for resistance, categorizing resistance mechanisms (antibiosis, antixenosis, tolerance), breeding for resistance traits, and integrating resistant varieties into pest management programs.^95^

Challenges include the genetic difficulty of transferring resistance traits into commercial crops, especially when dealing with polygenic rather than monogenic resistance.^96^ Moreover, resistant cultivars may have lower yields due to the resource costs of maintaining resistance or linkage drag, where resistance genes are linked to undesirable traits. Despite these limitations, HPR remains a successful strategy against various insect pests, although breeding for complex resistance traits is still challenging due to limited mechanistic understanding.^96^

In summary, there has been an increase in the breeding of biofortification in recent years for the three critical micronutrients, iron (Fe), zinc (Zn), and provitamin A. Adding resistance to major diseases and insects seems to be effective in lowering nutritional losses.^97^ Within the context of the IPM, recently emerging digital technologies have shown significant promise in the field of insect monitoring and local pest control, particularly in preventing population outbreaks. These procedures may involve the application of bioelicitors or specific fertilizers to ensure that crops receive the necessary nutrients to maintain their health and resistance to pests in order to minimize any substantial reductions in either nutritional quality or productivity.^98^ In addition to the progress in genome editing techniques that has facilitated the development of innovative pest management approaches through the production of genetically modified insects, such as the utilization of the CRISPR/Cas9 gene-editing system.^99^

#### 5.2.2 Contribution of GMOs in Mitigating the Impact of Insect Pests on Crop Nutritional Quality

Genetically modified organisms (GMOs) play a crucial role in minimizing insect pest impacts on crop yield and nutritional quality. By introducing pest-resistant traits, such as *Bacillus thuringiensis* (Bt) toxins, GM crops like Bt maize and cotton reduce insect damage, helping maintain protein, starch, and mineral levels in crops.^100,101^ This pest resistance crops also indirectly lowers fungal infection and mycotoxin contamination, improving food safety.^102,103^ GM crops can be biofortified to enhance their nutritional value while retaining pest resistance, exemplified by Golden Rice, which is engineered for higher beta-carotene content.^104^ Moreover, integrating GM crops within Integrated Pest Management (IPM) strategies helps reduce the need for chemical pesticides, promoting environmental sustainability.^105,106^

Despite the benefits, challenges like the evolution of pest resistance to Bt toxins, potential negative impact on beneficial insects such as natural enemies and pollinators, in addition to the public concerns about GMOs persist.^107,108^

Emerging technologies such as RNA interference (RNAi) and CRISPR/Cas9 gene editing are being explored to enhance pest management and crop resilience.^109^

Overall, GMOs and emerging technologies contribute significantly to maintaining crop nutritional quality and food security, especially when combined with sustainable agricultural practices.

#### 5.2.3 Cultural Practices and Biological Control

Cultural practices and biological control strategies are critical to preserving the nutritional quality of agricultural products by mitigating the impact of insect pests without relying significantly on synthetic pesticides. Crop rotation, planting date, and intercropping all contribute to a less favorable habitat for pests, lowering their populations and preventing them from causing major crop damage. For example, rotating crops or alternate planting patterns disrupt pest life cycles and reduce pest populations, while intercropping service crops can diversify cropping systems that confuses pests, attracting more natural enemies, and reducing pest pressure on main crops.^110^ For example, the timing of wheat planting significantly influences both quality and insect damage, especially concerning the Hessian fly (HF). The optimal strategy to mitigate the threat of HF destruction is the use of resistant types alongside early planting dates.^18^ Also, the beneficial role of egg parasitoids in reducing Sunn pest *E. integriceps* Puton [Hemiptera: Scutelleridae] populations has been recognized in Syria, Iran, and Turkey. As a result, these countries have started mass rearing these natural enemies and releasing them in wheat fields. Numerous studies demonstrated that gluten quality was substantially superior and comparable to that of non-Sunn pest infested wheat when 1, 2, or 3 egg parasitoids *Trissolcus grandis* [Hymenoptera: Scelionidae] were present at Sunn pest densities of 2 and 4 insects/m^2^. Moreover, many investigations have demonstrated that medicinal plants, as ecosystem services, can significantly attract the Sunn pest egg parasitoid *T. grandis*. Cultivating coriander adjacent to wheat fields may aid in the conservation and enhancement of Sunn pest parasitoids.^111^

Overall, these sustainable practices jointly enhance a more resilient agricultural system that harmonizes pest management with the maintenance of crop nutritional quality. By minimizing pest pressure by these tactics, farmers may maintain the health of their crops and ensure that the nutritional quality of their produce is not compromised. Moreover, these approaches diminish dependence on chemical pesticides, which can adversely affect beneficial organisms and destroy the environment, rendering them essential elements of sustainable agriculture.

## 6 Limitations and Future Perspectives

This work identifies several limitations in the current research on the impact of insect pests on crop nutritional quality.

- Limited available data: as many studies tend to focus on yield loss rather than changes in nutritional quality, making it difficult to draw definitive conclusions.
- Variability in study conditions: Geographical location, climate, soil conditions, and crop management strategies affect the results and complicate the generalization of findings across different areas and settings.
- Interdisciplinary research gap: Effective pest management and the preservation of nutritional quality require the integration of entomology, plant science, nutrition, and agricultural technology.
- Inconsistent methodologies in experimental design, measurement techniques, and data collection contribute to discrepancies in results, hindering reliable comparisons and conclusions.
- Unknown long-term impacts of pest infestations on crop nutritional quality: Most research has focused on immediate or short-term effects, leaving the chronic implications of pest pressure unexamined.

Looking ahead, several promising future perspectives emerge from this review, highlighting potential areas for further complementary research and development.

- Advancement of integrated pest management (IPM): Continuous study and application of innovative IPM techniques can result in more sustainable and ecologically sound methods of mitigating losses in quality caused by insect infestation.
- Investigating the genetic basis of plants responses to insect infestation to develop crops with enhanced resistance combined with improved nutritional quality.
- Employing metabolomics and proteomic analysis to understand the biochemical pathways involved in the alteration of nutritional properties of a crop in response to insect pests’ damage.

## 7 Conclusion

In conclusion, this review emphasized the detrimental effects of insect damage on the nutritional value of agricultural production, including changes in carbohydrate and fat content, increased protein content, and reduced essential amino acids and fiber levels. Reducing quality losses from insect pest infestations requires a multidimensional approach, incorporating effective control techniques, advanced breeding programs, and novel pest management strategies. The nutritional value of crops is seriously threatened by field insect pests, a topic that has not received much attention in research, which highlights the importance of developing comprehensive strategies that include understanding the nature of nutritional losses and the specific damage mechanisms under climate changes. The utilization of genome editing techniques for pest management and the adoption of integrated pest management including the use of resistant/tolerant cultivars to insect pests, use of biofortified crops, promoting crop diversity and rotation, enabling early detection and intervention, employing targeted control measures seem very effective to limit the pest pressure. The synergy between these techniques has the potential to reduce the impact of insect pests on the nutritional content of crops while preserving beneficial organisms through reducing pesticide application. These combined efforts help ensure that crops remain healthy and nutritionally valuable for human consumption.
